# A Review on the Role of SNCA Gene in Neurodegenerative Diseases

**DOI:** 10.7759/cureus.69450

**Published:** 2024-09-15

**Authors:** Afrarahamed Jahabardeen, Nirenjen S, Narayanan J, Chitra V

**Affiliations:** 1 Pharmacy/Pharmacology, Sri Ramaswamy Memorial (SRM) College of Pharmacy, SRM Institute of Science and Technology, Chennai, IND

**Keywords:** alzhiemer’s disease, gene variations, neurodegeneration, parkinson' s disease, snca, α-synuclein

## Abstract

Gene variations significantly impact the development of neurodegenerative disorders, particularly Alzheimer's disease (AD) and Parkinson's disease (PD). In AD, which is marked by amyloid-beta (Aβ) plaques and tau tangles, key genetic contributors such as amyloid beta precursor protein (*APP*), presenilin (*PSEN1*), and presenilin 2 (*PSEN2*) play a significant role in early-onset familial AD due to their influence on Aβ accumulation. PD, marked by dopaminergic neuron degeneration and Lewy body formation, is associated with mutations in the *SNCA* gene encoding alpha-synuclein (α-Syn), as well as other genes such as leucine-rich repeat kinase 2 (*LRRK2*), Parkin RBR E3 ubiquitin-protein ligase (*PARK2*), PTEN-induced kinase 1 (*PINK1*), and protein deglycase (*DJ-1*). Genome-wide association studies have identified genetic variants in apolipoprotein (*APOE*)* *and *SNCA* that increase disease risk. Alpha-synuclein, a protein involved in synaptic function, misfolds and aggregates into toxic forms in neurodegenerative diseases. Aggregates disrupt neuronal functions and propagate in a prion-like manner, with *SNCA* mutations exacerbating α-Syn aggregation and disease severity. Alpha-synuclein levels in skin, serum, cerebrospinal fluid, and plasma distinguish PD patients from healthy patients, demonstrating biomarker potential for diagnosis and therapeutic strategies. Furthermore, α-Syn*’s* presence in neural crest-derived tissues from PD patients and melanoma patients suggests shared pathophysiological features. Ongoing research into *SNCA* and α-Syn is crucial for advancing diagnostics and therapeutics for neurodegenerative diseases.

## Introduction and background

**Figure 1:**
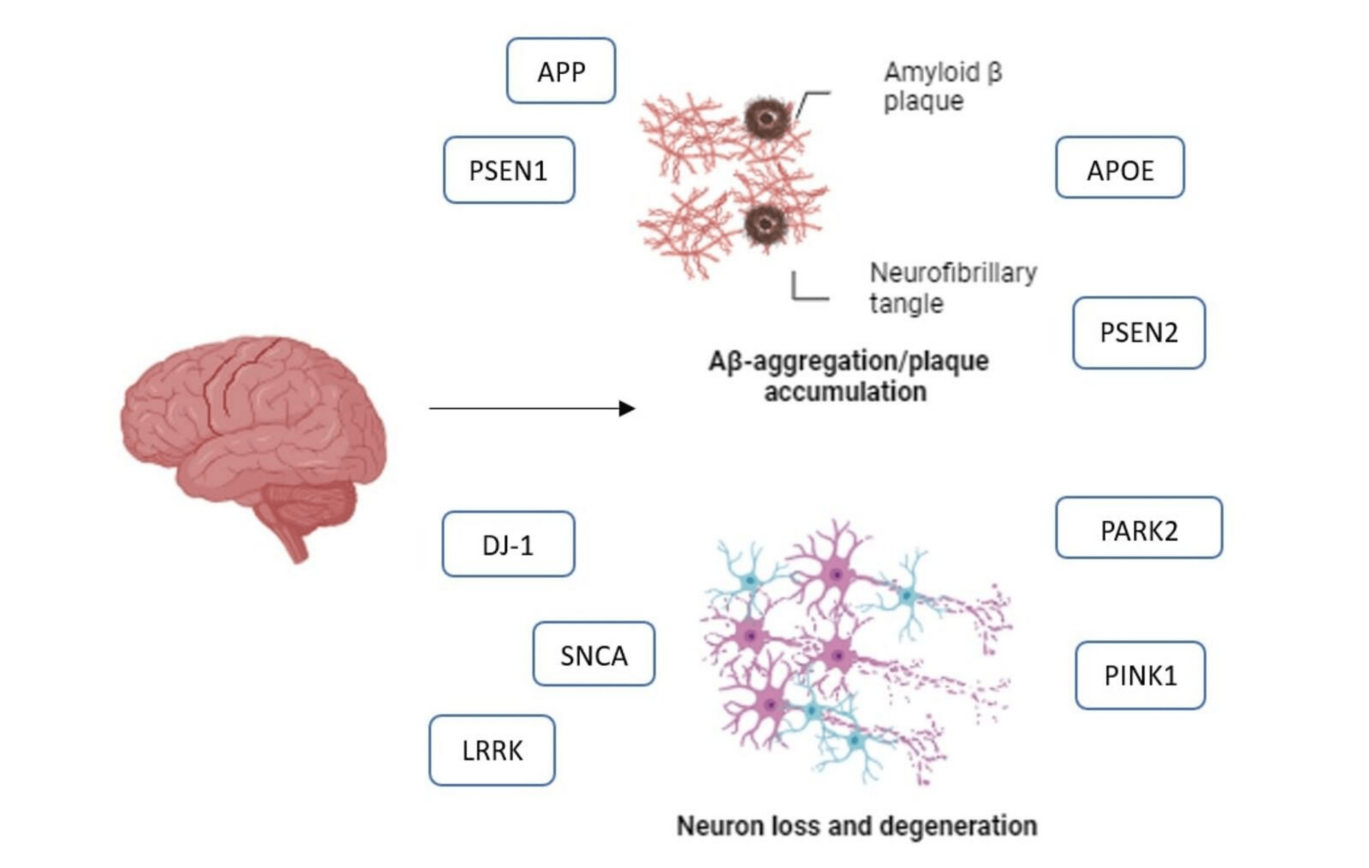
Gene variants in neurodegenerative conditions, with an emphasis on Alzheimer's disease and Parkinson's disease. A brain with two pathways shows various genes involvement in neurodegenerative processes. The primary pathway involves the *APP, PSEN1, APOE, and PSEN2*. This sequence of molecular events leads to the deposition of amyloid β plaques and the formation of neurofibrillary tangles. The process involves *DJ-1, SNCA, LRRK2, PARK2, *and *PINK1*, all of which contribute to neuronal loss and degeneration. The figure is illustrated by the author.

Genetic variations significantly contribute to the development and progression of neurodegenerative diseases, providing valuable insights into underlying mechanisms and potential therapeutic approaches. In Alzheimer's disease (AD), characterized by the presence of amyloid-beta (Aβ) plaques and tau protein tangles, critical genetic factors include the amyloid precursor protein (*APP*), presenilin 1 (*PSEN1*), presenilin 2 (*PSEN2*), and apolipoprotein E (*APOE*). These genes, particularly in cases of early-onset familial AD, influence the synthesis and accumulation of Aβ. In Parkinson's disease (PD), which is marked by the loss of dopaminergic neurons and the formation of Lewy bodies, specific monogenic forms are linked to mutations in genes such as synuclein alpha (*SNCA*), leucine-rich repeat kinase 2 (*LRRK2*), parkin *RBR E3* ubiquitin-protein ligase (*PARK2*), *PTEN*-induced putative kinase 1 (*PINK1*), and protein deglycase (*DJ-1*) [1]. Genome-wide association studies (GWAS) have uncovered genetic variants linked to AD and PD, showcasing the intricate genetic architecture of these conditions, as illustrated in Figure 1.

Notable risk variants, such as those in *APOE*, *SNCA*, and *MAPT *(microtubule-associated protein tau), have increased the likelihood of developing these diseases. GWAS works by analyzing millions of single-nucleotide polymorphisms (SNPs) throughout the genome to identify connections with disease susceptibility, offering crucial insights into the underlying mechanisms of these disorders and guiding the development of potential therapeutic strategies [2]. The *SNCA* gene is particularly significant in both PD and dementia with Lewy bodies (DLB) and AD, though secondary, and underscores the intricate interplay of genetic factors in neurodegenerative conditions. This complex genetic architecture emphasizes the need for continued research into genetic variations, particularly focusing on the *SNCA* gene, to improve the understanding and treatment of PD. Understanding the *SNCA* gene's contributions to these conditions is crucial for developing more targeted and effective therapeutic strategies. This review will delve into the pivotal role of *SNCA* in neurodegenerative diseases, exploring its pathophysiological mechanisms, clinical implications, and potential as a therapeutic target [3].

## Review

Pathophysiology of *SNCA* in neurodegenerative diseases

**Figure 2:**
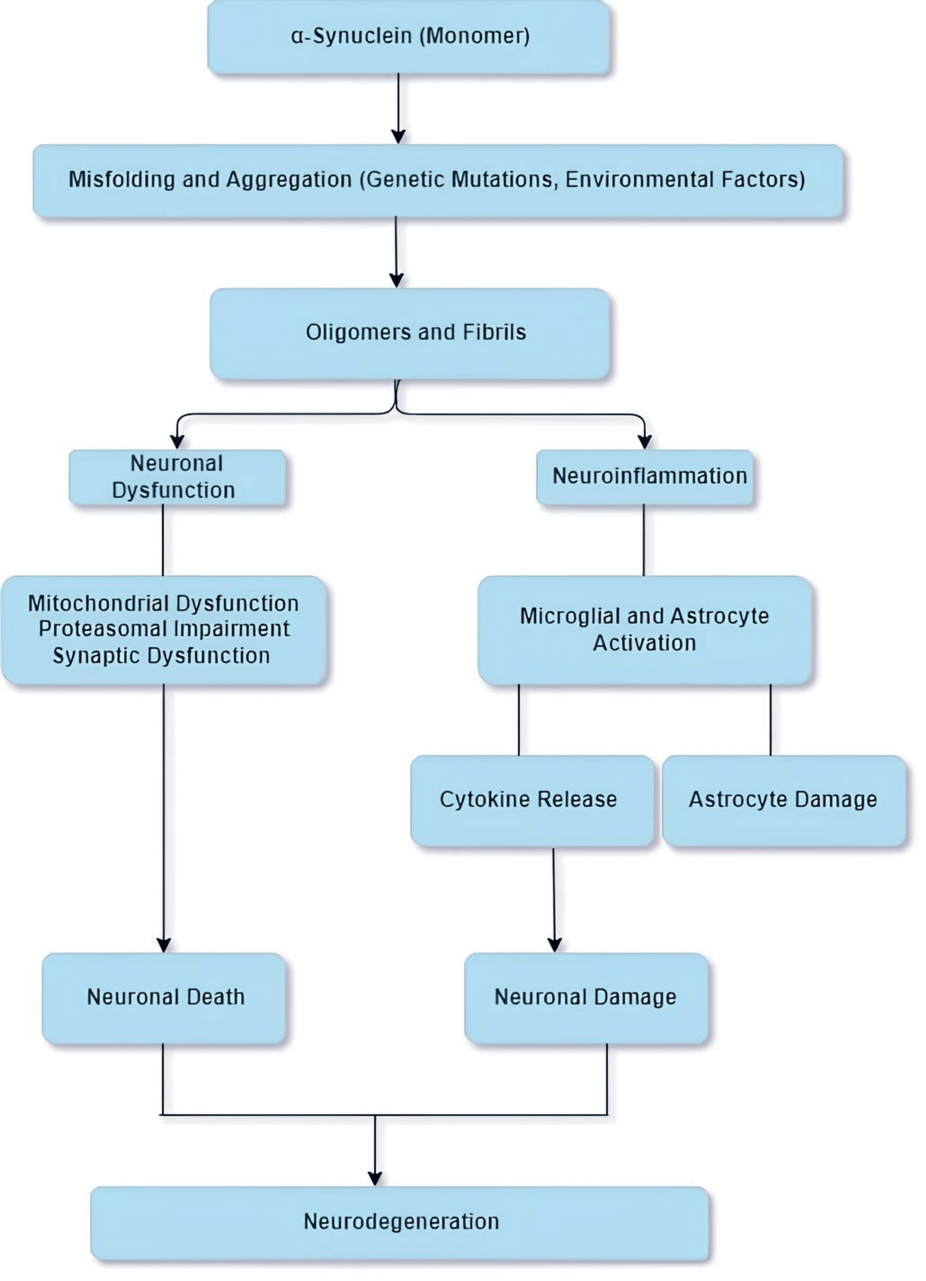
Pathophysiology of SNCA gene mechanism The pathological process of alpha-synuclein misfolding and aggregation leads to oligomer and fibril formation. This process causes neuronal dysfunction (via mitochondrial and proteasomal impairment) and neuroinflammation (via microglial and astrocyte activation), ultimately resulting in neuronal death and damage, contributing to neurodegeneration. The figure is illustrated by the author. SNCA, synuclein alpha

Alpha-synuclein (α-Syn), a protein encoded by the *SNCA* gene, is critically involved in the development of neurodegenerative disorders termed “synucleinopathies.” These diseases encompass PD, DLB, and multiple system atrophy. In healthy neurons, α-Syn is predominantly found in the presynaptic terminals that regulate neurotransmitter release and synaptic function. Structurally, it comprises three regions: the N-terminal, which is important for membrane binding; the non-amyloid-beta component (NAC), which is crucial for aggregation; and the C-terminal, which has neuroprotective properties [4]. Pathologically, *SNCA* misfolds and aggregates into oligomers and fibrils, forming Lewy bodies and neurites. These aggregates disrupt cellular functions such as synaptic transmission, mitochondrial function, and proteasomal degradation, leading to neuronal dysfunction and death [5]. Additionally, *SNCA* aggregates propagate in a prion-like manner, spreading between cells and inducing further misfolding. The aggregates also activate microglia and astrocytes, causing chronic neuroinflammation that exacerbates neuronal damage. Mutations in the *SNCA* gene (e.g., A53T, A30P, E46K) and gene multiplications are linked to familial forms of PD and increase the tendency of α-Syn to aggregate. The combination of direct neuronal impairment and indirect damage through inflammation leads to the progressive neurodegeneration characteristic of synucleinopathies [6], as shown in Figure 2.

Clinical information and therapeutic approaches on the *SNCA* gene

Rodríguez-Leyva et al. performed skin biopsies to detect α-Syn inclusions. The biopsies were analyzed using immunohistochemistry (IHC) and immunofluorescence (IF) to detect and quantify *SNCA*. Patients were divided into those with PD and those with atypical parkinsonism (AP), including both neurodegenerative and secondary causes. The skin samples were analyzed using light microscopy and confocal microscopy to identify and quantify *SNCA*-positive cells in the epidermis, pilosebaceous units (PSU), and eccrine glands. Significant α-Syn positivity was found in 60% of epidermal and PSU cells in PD patients, while it was minimal in AP patients (median of 6.9% in the epidermis, 7.7% in PSU, 0% in eccrine glands). The healthy patient group showed no immunoreaction. Confocal microscopy confirmed that *SNCA *inclusions were predominantly juxtanuclear in PD patients, consistent with findings from light microscopy. Further research and confirmation of these findings could lead to the development of new diagnostic protocols [7].

Smith et al. examined serum levels of α-Syn and anti-α-Syn antibodies in patients with PD, those with atypical Parkinson syndromes (APS), those with idiopathic rapid eye movement sleep behavior disorder (RBD), and healthy patients. Serum samples were collected, processed, and stored before analysis. Alpha-synuclein levels were measured using an enzyme-linked immunosorbent assay (ELISA), with serum diluted and tested in multiple copies. Anti-α-Syn antibodies were also detected using ELISA in various tests. A comparison of serum levels between groups suggests the potential of anti-α-Syn antibodies as both a biomarker and a neuroprotective agent [8].

Nishioka et al. reported a patient with the α-Syn p.A53T variant showing PD symptoms at the age of 42 years. The study involved a genetic analysis of a patient with the *SNCA* p.A53T variant using targeted gene panel sequencing. DNA was extracted from the patient and confirmed using Sanger sequencing. Haplotype analysis compared the patient’s variant with that from an Italian case using microsatellites. Brain pathology was assessed through immunohistochemical staining and microscopic examination, including anti-phosphorylated α-Syn and tau antibodies. Over time, his condition worsened, presenting with bradykinesia, rigidity, scoliosis, and cognitive decline, confirmed by reduced scores on cognitive tests. Magnetic resonance imaging (MRI) scans initially showed normal results but later revealed atrophy in the hippocampus and temporal lobes. Genetic analysis identified a rare *SNCA* c.157G>A p.A53T variant, and haplotype analysis showed differences from an Italian family with the same mutation. Neuropathological examination revealed severe neuronal loss, Lewy bodies, and astrogliosis, with Braak stage VI Lewy pathology. The patient responded well to levodopa but eventually died at age 52 due to pneumonia and multiple organ failure. Comparatively, similar cases with the *SNCA* p.A53T variant showed early onset, rapid progression, and severe cognitive decline, with an average onset age of 43.2 years and death by 53.3 years. Findings demonstrate the severe impact of the *SNCA* p.A53T variant [9].

Byers et al. used induced pluripotent stem cell (iPSC) technology to study early-onset PD in a patient with an *SNCA *gene triplication. In this study, primary cell lines were derived from human dermal fibroblasts of individuals with* SNCA *triplication and healthy controls, cultured in DMEM/F12 with fetal bovine serum. Mouse embryonic fibroblasts were prepared from CF-1 mice for use as feeder layers. Retroviral vectors were used to reprogram fibroblasts into iPSCs. Differentiation into dopaminergic neurons was directed using specific growth factors and media. A 48-year-old male with an *SNCA *triplication presented with early-onset PD at age 38, initially experiencing symptoms such as fatigue, tremors, and decreased dexterity, which progressed to mild cognitive and psychiatric symptoms by 2008. The iPSCs were generated from the patient's and an unaffected sibling's dermal fibroblasts and maintained human embryonic stem cell-like properties for more than 30 passages. These iPSCs were differentiated into dopaminergic neurons, with no significant difference in the number of tyrosine hydroxylase (TH)-positive neurons between patient and control lines. However, neurons derived from the patient exhibited elevated *SNCA* expression (1.44 times higher than Ctrl2 and 7.17 times higher than H9, p = 0.013) and a twofold increase in α-Syn protein. Additionally, patient-derived dopaminergic neurons showed increased expression of oxidative stress and protein aggregation-related genes (1.5- to fourfold higher) and were more susceptible to oxidative stress-induced cell death, with significantly higher numbers of caspase-3 positive cells under hydrogen peroxide challenge [10].

Mejía et al. suggested using skin and blood samples from relapsing-remitting multiple sclerosis (RRMS) patients and healthy controls to measure α-Syn levels. Alpha-synuclein levels were measured in skin tissue using immunohistochemistry and in peripheral blood mononuclear cells (PBMCs) with flow cytometry. Immunohistochemistry involved formaldehyde fixation, paraffin embedding, and staining using anti-α-Syn antibodies, followed by digital analysis for α-Syn positive. Flow cytometry detected α-Syn in PBMCs with specific antibodies and fluorescence detection [11].

Bousiges et al. highlighted a study where patients were recruited from the AlphaLewyMA protocol, involving a tertiary memory clinic. They were evaluated for DLB and AD using detailed clinical assessments, neuropsychological evaluations, blood tests, MRI, and cerebrospinal fluid (CSF) analysis. CSF samples were collected via lumbar puncture and analyzed for various biomarkers, including amyloid-beta 42 (Aβ42), amyloid-beta 40 (Aβ40), total tau (t-tau), phospho-tau181, and total α-Syn using ELISA. The study included patients with DLB, AD, and a healthy patient group, with additional subgroups for prodromal and demented stages of each condition [12].

Chahine et al. conducted a study requiring at least two types of biofluids and two tissue types for inclusion. Tissue processing involved formalin fixation, paraffin embedding, and staining for α-Syn, with slides reviewed by neuropathologists. Biofluids were processed and stored for analysis of α-Syn levels using ELISA. Feasibility and safety were assessed through metrics such as specimen adequacy and adverse event rates, with statistical analyses performed using SAS 9.4 to compare PD and healthy control groups and explore predictors of sample adequacy and adverse events. This study highlighted α-Syn's importance as a biomarker for PD [13].

Tokuda et al. used ELISAs to measure levels of total and oligomeric α-Syn in CSF from patients with PD and controls. Total α-Syn was quantified using a sandwich ELISA with chemiluminescence detection, which involved capturing α-Syn with a monoclonal antibody and detecting it with a polyclonal antibody linked to horseradish peroxidase. For oligomeric α-Syn, a similar ELISA was employed, but it was designed to specifically detect high-molecular-weight oligomers. Statistical analyses included comparisons using Mann-Whitney U tests and receiver operating characteristic curves to determine diagnostic cutoffs [14].

Chang et al. measured plasma and serum α-Syn levels in PD patients and healthy patients using an immunomagnetic reduction (IMR) assay. Blood samples were collected, processed to obtain plasma or serum, and analyzed using magnetic nanoparticles functionalized with α-Syn antibodies. The alternative-current magnetic susceptibility of the samples, which correlates with α-Syn concentration, was measured to quantify the protein levels [15].

Rodríguez-Leyva et al. analyzed skin biopsies from patients with PD, melanoma, nevi, skin tags, and healthy patients to investigate the presence of α-Syn and tyrosinase. Biopsies were collected from various centers and subjected to immunohistochemical staining for α-Syn and tyrosinase, with samples processed by fixation in paraffin, slicing, and staining. The presence of α-Syn was quantitatively assessed using image analysis software, comparing its distribution and intensity across different tissue types. Immunofluorescence was employed to further examine co-expression patterns of α-Syn and tyrosinase in melanoma cases [16]. Statistical analysis was conducted to compare the immunopositivity of α-Syn between groups, and these studies were clearly cited in Table 1.

**Table 1:** Clinical Information and Therapeutic Approaches on SNCA Gene: The table illustrates the various studies discussing the Clinical Information and Therapeutic Approaches on SNCA Gene and are cited accordingly Aβ42, amyloid-beta 42; Aβ40, amyloid-beta 40; α-Syn, alpha-synuclein; AD, Alzheimer's disease; AP, atypical parkinsonism; APS, atypical parkinsonian syndromes; CSF, cerebrospinal fluid; DLB, dementia with Lewy bodies; iPSC, induced pluripotent stem cells; MDS, Movement Disorder Society; MS, multiple sclerosis; PD, Parkinson's disease; p-tau, phosphorylated tau; RBD, rapid eye movement sleep behavior disorder; SNCA, synuclein alpha gene; t-tau, total tau

Study Title	Patient Groups	Biomarker(s) Measured	Key Findings	Therapeutic Approaches
α‐Synuclein inclusions in the skin of Parkinson's disease and parkinsonism [7]	20 healthy patients, 34 PD patients, 33 AP patients	α-Syn in skin biopsies	α-Syn inclusions were detectable in skin biopsies, distinguishing PD patients from healthy controls and AP patients.	Diagnostic approaches focus on early PD diagnosis by detecting α-Syn in accessible tissues such as skin, offering a less invasive method compared to traditional techniques.
α-Synuclein and anti-α-synuclein antibodies in Parkinson's disease, atypical Parkinson syndromes, REM sleep behavior disorder, and healthy controls [8]	PD (n = 14), APS (n = 11), RBD (n = 10), healthy patients (n = 9)	Serum α-Syn, anti-α-Syn antibodies	Power analysis indicated that detecting a 25% difference in α-Syn levels requires 236 samples per group, while a 50% difference requires 73. This highlights the challenge of using serum α-Syn as a biomarker for PD.	Potential neuroprotective effects of anti-α-Syn antibodies as therapeutic strategies for PD and synucleinopathies.
Pathological findings in a patient with alpha-synuclein p.A53T and familial Parkinson's disease [9]	Case study of a patient with α-Syn p.A53T variant. Symptoms started at 42 years and included cognitive decline and Lewy pathology post-mortem	p.A53T variant in the SNCA gene	The p.A53T variant in SNCA was identified, with severe Lewy pathology correlating with clinical symptoms	The initial response to levodopa-carbidopa, electroconvulsive therapy for psychosis, and supportive care in later stages are related to SNCA gene variants, influencing PD symptoms and progression.
SNCA triplication Parkinson's Patient's iPSC-derived DA neurons accumulate α-synuclein and are susceptible to oxidative stress [10]	Early-onset PD patient with SNCA triplication; symptoms include tremor, cognitive issues, sleep apnea	α-Syn in iPSC-derived neurons	Increased α-Syn accumulation and oxidative stress in neurons, modelling PD pathology.	Using patient-specific iPSC-derived neurons to model disease mechanisms, targeting protein aggregation and oxidative stress for therapy development.
Low levels of alpha-synuclein in peripheral tissues are related to clinical relapse in relapsing-remitting multiple sclerosis: a pilot cross-sectional study [11]	Skin samples and peripheral blood were collected from 34 healthy patients and 23 MS patients to test α-Syn levels. In the RRMS group, 15 individuals were in remission, whereas 8 were in the relapse phase.	α-Syn levels during remission and relapse.	α-Syn levels were lower in relapsing MS than in the other groups, both in the positive area (p = 0.021), and staining intensity (p = 0.004) were observed during relapses, suggesting its potential as a recurrence marker.	α-Syn as a marker for inflammation and relapse in MS. Intravenous methylprednisolone treatment impacts immune activity.
Differential diagnostic value of total alpha-synuclein assay in the cerebrospinal fluid between Alzheimer’s disease and dementia with Lewy bodies from the prodromal stage [12]	Study includes 166 patients categorized into six groups: healthy patients, prodromal DLB patients, demented DLB patients, prodromal AD patients, demented AD patients, and AD/DLB patients.	CSF α-Syn, t-Tau, p-Tau, Aβ42, Aβ40	Differences in α-syn levels helped differentiate DLB from AD, aiding in diagnosis.	To improve diagnostic accuracy, use a multimodal approach that includes clinical criteria (e.g., McKeith's), biomarker analysis (e.g., CSF α-syn and Alzheimer's biomarkers), and neuroimaging (e.g., DaT-SPECT and MRI), focusing on diagnostic methodologies using biomarkers for differential diagnosis of DLB and AD to tailor therapeutic strategies.
Feasibility and safety of multicenter tissue and biofluid sampling for α-synuclein in Parkinson’s disease: the systemic synuclein sampling study (S4) [13]	Evaluates α-Syn in multiple tissues and biofluids of PD patients and healthy patients. PD patients are divided into early, moderate, and advanced groups.	α-Syn in multiple tissues and biofluids.	Widespread α-Syn pathology observed in PD patients, correlated with disease stage.	Emphasizes the importance of α-Syn as a biomarker for diagnosis, disease progression, target engagement, and therapeutic efficacy in PD.
Detection of elevated levels of α-synuclein oligomers in CSF from patients with Parkinson disease [14]	32 PD patients and a control group with various neurological disorders. Measures α-Syn oligomers in CSF.	α-Syn oligomers in CSF.	Higher levels of α-Syn oligomers in PD patients suggest its potential as a diagnostic biomarker.	Aims to develop a reliable biomarker for PD diagnosis based on α-Syn oligomer levels in CSF.
Plasma and serum alpha-synuclein as a biomarker of diagnosis in patients with Parkinson's disease [15]	Study with 48 PD patients and 40 healthy patients. Diagnosis based on the MDS criteria. Clinical data collected.	α-Syn in plasma and serum	PD patients had considerably greater levels of plasma α-Syn, which supports its utility as a biomarker.	Focus on α-Syn levels as a biomarker for PD diagnosis and progression, guiding treatment decisions.
The presence of alpha‐synuclein in skin from melanoma and patients with Parkinson's disease [16]	Compares α-Syn in neural crest-derived tissues from PD patients, melanoma, nevi, skin tags, and healthy skin.	α-Syn in neural crest-derived tissues.	α-Syn was detectable in PD patient tissues, suggesting a link between PD and melanoma.	Suggests shared pathophysiology between PD and melanoma. Targeting α-Syn aggregation and addressing oxidative stress and inflammation as potential therapeutic strategies.

Complications and challenges: targeting *SNCA* for therapeutic intervention

Drug Delivery Especially Across the Blood-Brain Barrier

Administration of certain therapeutic agents to the CNS is difficult because the blood-brain barrier inhibits most drugs from penetrating the brain mass. This is more of an issue when therapies are designed for delivery into α-Syn aggregates, which have to be delivered to neuronal cells. Scientists are also trying to identify ways to counter this problem such as nanoparticles, focused ultrasound, and delivery systems. Nevertheless, such approaches must be better targeted to minimize effectivity and possible side effects.

Specificity and Off-Target Effects

Since α-Syn plays a normal physiological role, drugs intended to alter the aggregation or function of α-Syn must be very selective. It has also been established that non-specific interactions may cause side effects and toxicity. The creation of small molecules or peptides or high selectivity antibodies that will bind α-Syn selectively and not other proteins is very desirable. Furthermore, identifying the unique structures of α-Syn (for instance, oligomers compared to fibrils) promotes a better strategy to focus on [17]. 

Therapeutic Modalities

Other explored approaches include gene knockdown (for example, RNA interference), immune modulation, and small molecules. Each has its problems associated especially with delivery, effectiveness, and safety. Hybrid of the various therapeutic methods or application of more innovative techniques such as the Clustered Regularly Interspaced Short Palindromic Repeats (CRISPR/Cas9) system for gene manipulation may provide a better solution. Other clinical trials will reveal the optimal modalities that could be implemented.

Genetic Heterogeneity

The *SNCA* gene presents different types of mutations, for example, point mutations such as A53T and A30P, and copy number variations such as duplications or triplications of the gene. Such variations can give rise to diverse disease characteristics types and progressiveness of the sickness. Hence, individualized management strategies have to be employed to address this variability. Genetic screening and patient stratification make it easier to approach patients, but the intricacies of such variants make it a difficult task [18].

Phenotypic Diversity

Patients with different *SNCA* mutations or CNVs may present with varying clinical symptoms and disease severities. This variability can complicate diagnosis and treatment strategies. Understanding the relationship between specific genetic variants and clinical outcomes is essential for developing targeted therapies. Large-scale studies and detailed phenotyping can help in elucidating these relationships.

Diagnostic Challenges

The presence of multiple *SNCA* variants and their diverse effects can make accurate diagnosis difficult. Some variants may lead to atypical or less recognizable disease forms. Improved diagnostic tools and criteria are needed to better classify and diagnose *SNCA*-related disorders. Enhanced genetic and clinical assessments can aid in more accurate disease characterization [19].

By targeting the *SNCA* gene for therapeutic intervention and focusing on the development and validation of α-Syn as a biomarker for early diagnosis and disease progression, researchers identify novel therapeutic targets. This is achieved by studying the effects of *SNCA* mutations and gene duplications on α-Syn aggregation [20].

Discussion

The *SNCA *gene, encoding the protein α-Syn, has been extensively studied due to its significant role in neurodegenerative disorders, particularly PD and DLB. This review highlights the pivotal contributions of *SNCA *mutations and gene duplications in the pathogenesis of these diseases and discusses current advancements in diagnostic and therapeutic strategies. Hence, the review indicates the centrality of α-Syn in the development of synucleinopathies. Mutations such as A53T, A30P, and E46K, as well as gene multiplications, lead to an increased propensity for α-Syn aggregation, forming toxic oligomers and fibrils. These aggregates disrupt various cellular processes, including synaptic transmission, mitochondrial function, and proteasomal degradation, ultimately resulting in neuronal dysfunction and death. Additionally, the prion-like propagation of α-Syn aggregates exacerbates neurodegeneration by spreading pathology between cells.

Several investigations have demonstrated the diagnostic potential of α-Syn. Elevated levels of α-Syn in CSF and serum have been suggested as biomarkers for PD. Innovative diagnostic techniques, such as immunohistochemistry detection of α-Syn in skin biopsies, offer less invasive alternatives to traditional methods, facilitating earlier and more accurate diagnosis. Targeting α-Syn aggregation and its consequences is a viable therapeutic approach. Induced pluripotent stem cell (iPSC) models have provided valuable insights into disease mechanisms and potential therapeutic targets. These models have demonstrated the efficacy of strategies aimed at reducing oxidative stress and mitigating protein aggregation. Despite significant progress, several gaps remain in our understanding of *SNCA*-related pathogenesis and its broader implications. Detailed studies on the molecular mechanisms driving α-Syn aggregation and propagation are essential. Understanding the interplay between genetic mutations, environmental factors, and cellular stress responses will aid in identifying critical intervention points.

Recent biomarker advancements have expanded beyond traditional CSF and serum α-Syn measurements. New methods include detecting α-Syn in saliva and plasma, which offer non-invasive diagnostic options. Additionally, immunohistochemistry of α-Syn aggregates in skin biopsies provides a promising alternative to invasive procedures. Neurofilament light chain levels, while not specific to α-Syn, are also emerging as a useful marker for general neurodegeneration. These developments enhance early diagnosis, track disease progression, and assess therapeutic efficacy. The main approaches include designing small molecules, peptides, and peptidomimetics that directly inhibit α-Syn aggregation. Other therapies involve gene silencing, promoting intracellular clearance via autophagy, and immunotherapy aimed at degrading amyloid fibrils. Additionally, strategies such as receptor blocking to inhibit α-Syn spread and cell transplantation are under exploration. Although these therapies are in various stages of development, no disease-modifying treatments are currently available. Investigating the links between α-Syn and other conditions, such as melanoma, may uncover shared pathophysiological mechanisms and potential therapeutic targets. Additionally, understanding α-Syn’s role in other neurodegenerative diseases could provide broader insights into common disease pathways. Utilizing patient-specific iPSC models to study individual variations in disease presentation and progression can lead to personalized therapeutic approaches. These models can also be used to screen for drug efficacy and toxicity, accelerating the development of targeted treatments. The* SNCA* gene and its product, α-Syn, are central to the pathogenesis of PD and DLB. Advances in understanding the molecular underpinnings of α-Syn aggregation and its impact on neuronal function have paved the way for novel diagnostic and therapeutic approaches. Continued research in this area holds promise for improving the diagnosis, treatment, and overall outcomes for patients suffering from these debilitating disorders.

## Conclusions

The *SNCA* gene, which encodes α-Syn, is central to neurodegenerative disorders such as PD and DLB. *SNCA* mutations and gene duplications cause abnormal α-Syn aggregation, resulting in neuronal dysfunction and neurodegeneration. Advances in diagnostic techniques, including the detection of α-Syn in skin biopsies and CSF, offer promising avenues for early diagnosis and disease monitoring. Additionally, elevated α-Syn levels in serum and CSF suggest its potential as a biomarker for PD. Therapeutic strategies targeting α-Syn aggregation and related neuroinflammation are being explored, with promising results from iPSC-derived neuron models. The connections between α-Syn and other conditions, such as melanoma, also highlight its broader relevance. Continued research in *SNCA* and α-Syn is crucial for developing effective diagnostics and therapies, ultimately improving patient outcomes in neurodegenerative diseases.
